# Synthesis of Novel Hyperbranched Polybenzo-Bisthiazole Amide with Donor–Acceptor (D-A) Architecture, High Fluorescent Quantum Yield and Large Stokes Shift

**DOI:** 10.3390/polym9080304

**Published:** 2017-07-25

**Authors:** Xiaobing Hu

**Affiliations:** College of Chemistry and Chemical Engineering, Baoji University of Arts and Sciences, Shaanxi Province Key Laboratory of Phytochemistry, Baoji 721013, Shaanxi, China; hxb.0917@stu.xjtu.edu.cn; Tel.: +86-917-356-6589

**Keywords:** hyperbranched polyamide, 1,3,5-tris(4-carboxyphenyl) benzene, fluorescence quantum yield, donor–acceptor architecture

## Abstract

Two novel highly fluorescent hyperbranched polybenzobisthiazole amides with a donor–acceptor architecture and large Stokes shift were rationally designed and synthesized. The chemical structures of the prepared hyperbranched polymers were characterized using Fourier Transform Infrared Spectroscopy (FTIR) analysis, Hydrogen Nuclear Magnetic Resonance (^1^H-NMR) analysis, and Gel Permeation Chromatography (GPC) analysis. These two polymers were soluble in dimethyl sulfoxide (DMSO) and *N*,*N*-dimethylformamide (DMF), and their DMSO and DMF solutions emitted strong green light (517–537 nm) with high quantum yields (QYs) and large Stokes shifts. Their relative fluorescence QYs in the DMSO solution were calculated as 77.75% and 81.14% with the Stokes shifts of 137 nm (0.86 eV) and 149 nm (0.92 eV) for HP–COOH and HP–NH_2_, respectively, using quinine sulfate as the standard. In the DMF solution, the QYs of HP–COOH and HP–NH_2_ were calculated as 104.65% and 118.72%, with the Stokes shifts of 128 nm (0.79 eV) and 147 nm (0.87 eV), respectively. Their films mainly emitted strong blue light with the maximum emission wavelengths of 436 nm and 480 nm for HP–COOH and HP–NH_2_, respectively. The Stokes shifts for HP–COOH and HP–NH_2_ films were 131 nm (0.42 eV) and 179 nm (0.86 eV), respectively. They are promising candidates for luminescent solar concentrators and blue light emitting materials.

## 1. Introduction

Over the past few decades, hyperbranched polymers have drawn continuous and considerable attention because of their unique molecular architecture, improved physical and chemical properties, and their broad range of applications, such as fluorescent probes [1,2,3,4,5], polymer coatings [6,7], Separation materials [8], drug or biomolecule carrier materials [9,10,11,12,13,14,15,16], and in optoelectronic materials and devices [17,18,19,20,21,22,23,24,25,26]. Compared to linear polymers, hyperbranched polymers have the obvious advantages of high solubility, little chain entanglement, low viscosity, good processability, tunable light emission, low crystallinity, and controllable thin film morphology [27,28]. They have highly branched architecture, and large numbers of functional terminal groups and nanoscale cavities, and they are superior to their dendritic counterparts because of their convenient “one-pot” synthesis and their potential for large-scale production [29]. Generally, two kinds of strategies are used to synthesize hyperbranched polymers: the AB_n_ (when *n* ≥ 2) strategy and the A_2_ + B_n_ (when *n* ≥ 3) strategy [30,31,32]. For the AB_n_ strategy, although gelation is easy to avoid in theory, the monomers are usually difficult to synthesize, because the molecules have two different reactive groups and they can undergo self-polymerization in some cases. The second strategy, A_2_ + B_n_, has certain merits, specifically, this strategy uses commercially available monomers and results in high-yield soluble hyperbranched polymers synthesized by controlling the polymerization conditions and quenching the reaction before the gel point [33]. Therefore, the A_2_ + B_n_ route has become an important method for synthesizing hyperbranched polymers with wide applications. 

Poly(*p*-phenylene benzobisthiazole) (PBZT) is a kind of aromatic heterocyclic conjugated polymer, which is well known for its excellent mechanical properties (high tensile strength and modulus), high chemical and thermo-oxidative stabilities [34], and large and ultrafast third-order nonlinear optics response [35,36,37,38,39]. PBZT is insoluble in common organic solvents because of the high degree of molecular rigidity and strong intermolecular interactions, making PBZT difficult to process and impeding further detailed and accurate studies on this type of conjugated polymer. Introducing hyperbranched structure into conjugated polymers is an effective strategy for solving this issue. In our previous work, hyperbranched structure was introduced into the PBZT backbone using the A_2_ + B_3_ method [39,40]. The prepared hyperbranched polybenzobisthiazoles were soluble in organic solvents, and their solutions emitted strong blue fluorescence [39,40].

**Scheme 1:**
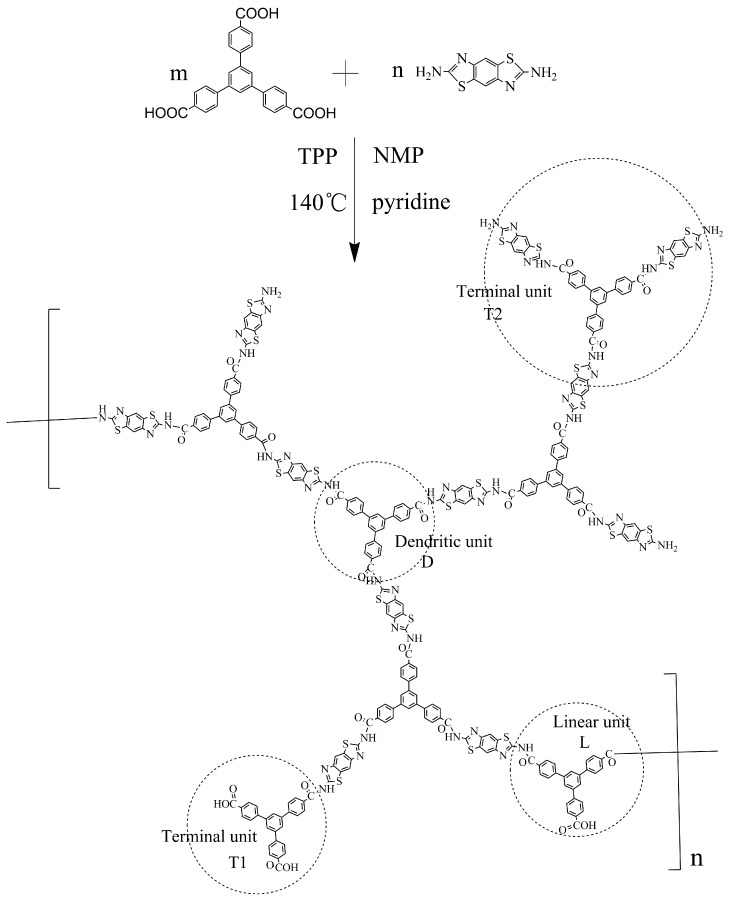
Synthetic route of hyperbranched polybenzobisthiazole amide.

In this contribution, two novel fluorescence hyperbranched polybenzobisthiazole amides with donor–acceptor (D-A) architecture were designed and synthesized using a direct polycondensation of 1,3,5-tris(4-carboxyphenyl) benzene (H_3_BTB, acceptor) with 2,6-diaminobenzo[1,2-d:4,5-d′]bisthiazole (DABBT, donor) via the A_2_ + B_3_ approach. The non-planar globular topological structures of the designed polymers make their chromophoric repeat units difficult to pack in a compact and ordered fashion. Consequently, the *π*–*π* stacking interaction in the polymer aggregates will be effectively suppressed, which is beneficial for a higher light emitting efficiency [3,41]. Furthermore, the donor–acceptor (D-A) architecture along the conjugated polymer backbones will imparts intramolecular charge transfer character, the highest occupied molecular orbital (HOMO) and the lowest unoccupied molecular orbital (LUMO) optical transition and larger effective Stokes shifts, hence endow adjustable optoelectronic properties and high solid-state fluorescence [42,43,44,45]. The synthetic approach is shown in Scheme 1. The condensation polymerization of DABBT and H_3_BTB was performed in two ways. Manner 1, DABBT and H_3_BTB were added in a mole feed ratio of 1:1 (m:n = 1:1), in which the carboxyl-terminated hyperbranched polyamide (HP–COOH) was generated. When the mole feed ratio of DABBT and H_3_BTB was 9:4 (Manner 2, m:n = 9:4), the amino-terminated hyperbranched polyamide (HP–NH_2_) was prepared. These two hyperbranched polyamides exhibit high fluorescence emission quantum efficiency because of the aromatic heterocyclic benzobisthiazole groups or H_3_BTB groups in their molecular structures which have displayed strong blue or green fluorescence in our previous studies, respectively [39,40,46]. These two polymers possess organo-solubility that allow for the facile and low-cost solution processing of an amorphous, highly luminescent thin film. Their relative QYs in DMSO and DMF solution were studied using quinine sulfate as the standard. The photophysical properties of the polymer thin films were also investigated.

## 2. Materials and Methods 

### 2.1. Starting Materials

DABBT was synthesized and purified using a slightly modified procedure reported in the literature [47,48]. The ^1^H-NMR and ^13^C-NMR spectra of DABBT are shown the Supplementary Materials (Figures S1 and S2). ^1^H-NMR (400 MHz, DMSO-*d*_6_, δ): 7.59 (s, 2H, Ar-H), 7.26 (s, 4H, –NH_2_). ^13^C-NMR (100 MHz, DMSO-*d*_6_, δ): 110.02, 129.62, 148.12, 165.28. H_3_BTB was synthesized using a two-step reaction as described in our previous work [40]. The ^1^H-NMR and ^13^C-NMR spectra of H_3_BTB are shown in the Supplementary Materials (Figures S3 and S4). ^1^H-NMR (400 MHz, DMSO-*d*_6_, δ): 8.03 (m, 15H, Ar-H), 12.99 (s, 3H, –COOH); ^13^C-NMR (100 MHz, DMSO-*d*_6_, δ): 167.57, 144.25, 141.16, 130.47, 130.33, 127.83, 125.98. *N*-Methyl-pyrrolidone (NMP), triphenyl phosphite (TPP), and pyridine (Py) were purchased from Sinopharm Chemical Reagent Co., Ltd. (Shanghai, China) without further purification. Quinine sulfate was purchased from Adamas Reagent, Ltd (Shanghai, China) and used as received.

### 2.2. Polymerization

The condensation polymerization of DABBT and H_3_BTB was performed in two manners with different mole feed ratios of the monomers as shown in Scheme 1. For the carboxyl-terminated hyperbranched polyamide (HP–COOH), the mole feed ratio of DABBT and H_3_BTB was 1:1, while for the amino-terminated hyperbranched polyamide (HP–NH_2_), the mole feed ratio of DABBT and H_3_BTB was 9:4. The specific reaction process was as follows. A 100 mL dry three-neck flask was purged with argon gas for 30 min. Then, 40 mL of N-methyl pyrrolidone (NMP), 3 mL of pyridine, 3.5 mL of triphenyl phosphite (TPP), and certain amounts of DABBT and H_3_BTB were added to the flask with continuous stirring and dry argon purging. After the mixture was dissolved, the reaction mixture was heated step wise to 140 °C the polymerization was performed at this temperature for about 10 h. The reaction system was then cooled naturally to room temperature; absolute methanol was added, and stirred for another 30 min. The precipitate was collected by vacuum filtration and washed with deionized water several times. Finally, the polymer was extracted with deionized water in a Soxhlet apparatus for 24 h and then dried at 90 °C for 48 h under reduced pressure. The yields of HP–COOH and HP–NH_2_ were 55.8% and 46.4%, respectively.

### 2.3. Measurements

^1^H-NMR and ^13^C-NMR spectra were recorded on a Varian 400 MHz NMR spectrometer using deuterated dimethyl sulfoxide (DMSO-*d*_6_) as the solvent and tetramethylsilane (TMS) as an internal reference. Fourier transform infrared (FTIR) spectra were measured on a Perkin Amelmer infrared spectrometer at room temperature. GPC measurements were performed on a Waters 1515 GPC system under 35 °C, DMF was used as the eluent with a flow rate of 1.0 mL/min. The molecular weights and molecular weight distributions of the polymers were calculated using narrow-molecular-weight polystyrene standards as reference. UV–Vis absorption spectra of polymers in DMSO and DMF solutions were recorded on a Shimadzu UV-2550 spectrometer (Shimadzu Co., Ltd., Kyoto, Japan) over a wavelength range of 250–800 nm. Wide-angle powder X-ray diffraction (XRD) patterns were recorded at room temperature on a Rigaku Ultima IV X-ray (Rigaku Corporation, Tokyo, Japan) diffraction system using Cu Kα radiation (λ = 0.15406 nm), at a scanning rate of 2°/s. Thermogravimetric analyses (TGA) were recorded on a Perkin-Elmer (Perkin-Elmer Co., Ltd., Massachusetts, the United States) TG/DTA under nitrogen flow with a heating rate of 10 °C/min. The temperature range was from 30 to 800 °C. Differential scanning calorimetry (DSC) analyses were performed on a TA Instruments DSC Q200 (TA Instrument Co., Ltd., New Castle, the United States) using sealed aluminum sample pans and sealed aluminum reference pans at a heating rate of 10 °C/min under a nitrogen atmosphere. The temperature range was from −80 to 400 °C. Fluorescence spectra were recorded on a Horiba scientific Fluoromax-4 spectrofluorometer (HORIBA Scientific, Edison, the United States) using spectroscopic grade DMSO and DMF as solvents at room temperature. The excitation and emission slit widths were 5 nm. All fluorescence emission spectra are blank-corrected and corrected for instrument-specific effects. The polymer films were prepared by solvent evaporation of polymer solution on the glass substrate. The relative QYs of the polymers were measured using quinine sulfate as the standard. Ф_FL_ of polymer films were measured by Horiba scientific Fluoromax-4 spectrofluorometer with an integral sphere.

## 3. Results and Discussion

### 3.1. NMR Analysis

**Figure 1:**
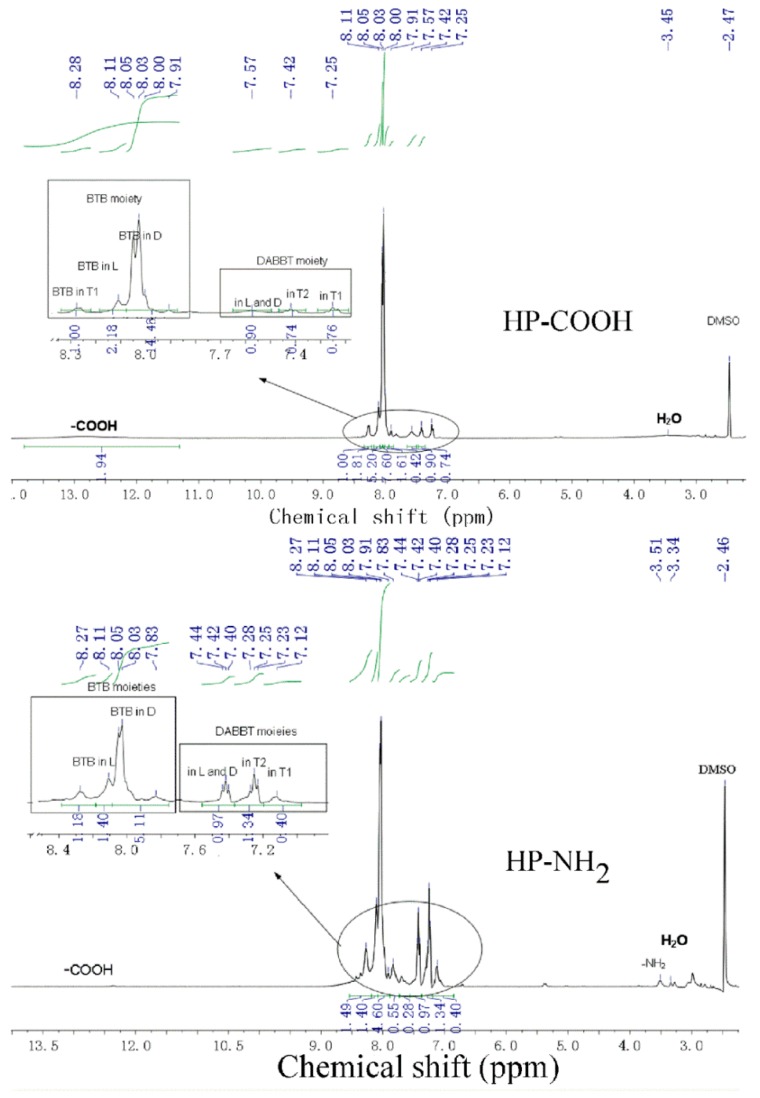
^1^H-NMR spectra of HP–COOH and HP–NH_2_.

The ^1^H-NMR spectra of HP–COOH and HP–NH_2_ in DMSO-*d*_6_ are shown in Figure 1. For HP–COOH and HP–NH_2_, the 1,3,5-trisubstitutedphenyl benzene (1,3,5-TPB) moiety acts as the branching point. All of the possible molecule structures of the obtained polymers (HP–COOH and HP–NH_2_) are shown in Scheme 1 with a focus on the functional groups bonded to the 1,3,5-TPB moiety. The number of remaining carboxylic groups (2, 1, or 0) or amino groups (1 or 0) of the 1,3,5-TPB moiety determines the assignment of the terminal unit (T_1_ or T_2_), linear unit (L_1_ or L_2_), and dendritic unit (D) [49] as shown in Scheme 1. When the number of remaining carboxylic groups or amino groups is two, the 1,3,5-TPB moiety belongs to T_1_ or T_2_. When the number of remaining carboxylic groups is one, the 1,3,5-TPB moiety is L_1_. When there are no carboxylic groups or amino groups on the 1,3,5-TPB moiety, it belongs to D. The peaks at about δ = 2.47, and 3.45 ppm are assigned to the protons of DMSO-d_6_ and to the absorbed water in the polymers, respectively. For HP–COOH, the peaks near δ = 7.25 and 7.42 ppm are assigned to the protons of the DABBT moieties in the T_1_ and T_2_ units (see Scheme 1), respectively. The peaks around δ = 7.57 ppm correspond to the protons of the DABBT moiety in the L and D units (see Scheme 1). The peaks near δ = 7.91, 8.00, 8.03, and 8.05 ppm are assigned to the protons of the 1,3,5-TPB moieties in the D units. The peaks around δ = 8.11 and 8.28 ppm correspond to the protons of the 1,3,5-TPB moieties in the L and T_1_ units, respectively. The broad peak from δ = 11.0 to 13.0 ppm is assigned to the –COOH end groups of the hyperbranched polyamide. For HP–NH_2_, the peaks near δ = 7.12 ppm are assigned to the protons of the DABBT moieties in the T_1_ units. The peaks near δ = 7.23, 7.25, and 7.28 ppm are assigned to the protons of the DABBT moieties in the T_2_ units. The peaks around δ = 7.40, 7.42, and 7.44 ppm correspond to the protons of the DABBT moiety in the L and D units. The peaks near δ = 7.83, 8.03, and 8.05 ppm are assigned to the protons of the 1,3,5-TPB moieties in the D units. The peaks around δ = 8.11 and 8.27 ppm correspond to the protons of the BTB moieties in the L and T_1_ units, respectively. The weak and broad peak from around δ = 11.0 to 13.0 ppm is assigned to the –COOH end groups in the polymer structure, which is much lower than that of HP–COOH.

The degree of branching (DB) is one of the most critical structural characteristics for hyperbranched polymers, which describes the structural perfection of hyperbranched polymers. DB was usually defined by Fréchet and coworkers as the following equation: [50]

(1) $$\mathrm{DB}\;=\;\frac{D\;+\;T}{D\;+\;T\;+\;L}$$

where D, T, and L refer to the number of dendritic, terminal, and linear units in the polymer, respectively. Generally, the values of D, T, and L can be determined by ^1^H-NMR measurement according to the integrated area of corresponding proton resonance peak. However, unfortunately, in this study, the relative amounts of D, T, and L were difficult to determine by ^1^H-NMR. As a result, it was hard to calculate the degree of branching (DB).

### 3.2. FTIR Analysis

The FTIR spectra of HP–COOH and HP–NH_2_ are shown in the Supplementary Materials (Figure S5). The absorptions at 1683, 1545, and 1282 cm^−1^ are attributed to the amide groups that derive from the reactions between the –COOH groups of H_3_BTB and the –NH_2_ groups of DABBT. Concretely, the signal at 1683 cm^−1^ is assigned to the stretching vibration of –C=O, the absorption at 1545 cm^−1^ is assigned to the bending vibration of –N–H, and the absorption at 1282 cm^−1^ is attributed to the stretching vibration of –C–N. The absorptions at 1608 and 1485 cm^−1^ are assigned to the stretching vibration of –C=C– of the aromatic ring. The absorption bands around 1720 and 1233 cm^−1^ are assigned to the stretching vibrations of –C=O and –C–O in –COOH groups [51] and indicate the presence of –COOH groups remaining in both HP–COOH and HP–NH_2_. The FTIR analysis and NMR analysis demonstrate that the hyperbranched polybenzobisthiazole amides were successfully synthesized.

### 3.3. GPC Analysis

**Figure 2:**
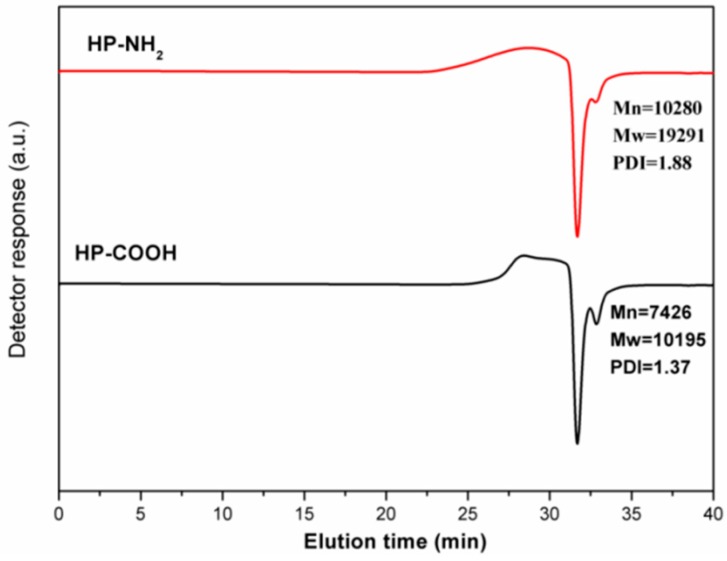
GPC curves of HP–COOH and HP–NH_2_.

The molecular weights and polydispersity index (PDI) values of the hyperbranched polybenzobisthiazole amides were measured using GPC with linear polystyrene as the standard, and the results are shown in Figure 2 and Table 1. The number-average molecular weight (M_n_), weight-average molecular weight (M_w_), molar mass at the peak maximum (M_p_), and the PDI value of HP–COOH are 7426 g/mol, 10,195 g/mol, 12,314 g/mol, and 1.37, respectively. For HP–NH_2_, the M_n_, M_w_, M_p_, and the PDI value are 10,280 g/mol, 19,291 g/mol, 11,035 g/mol, and 1.88, respectively (see Table 1). The PDI value of HP–COOH is relatively narrow which is uncommon for a hyperbranched polymer prepared via an A_2_ + B_3_ approach.

### 3.4. UV–Vis Absorption and Fluorescence Properties of HP–COOH and HP–NH_2_

**Figure 3:**
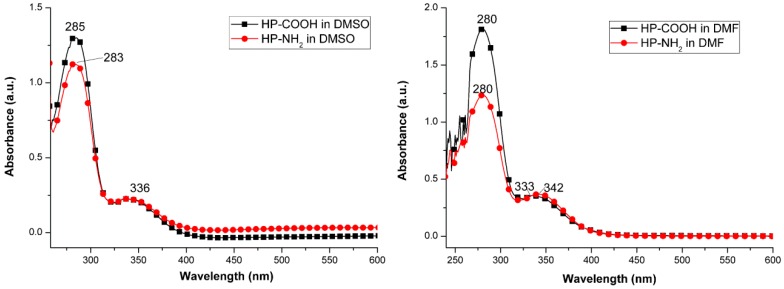
UV–Vis spectra of HP–COOH and HP–NH_2_ in: DMSO (**a**); and DMF (**b**) solutions.

Figure 3a,b shows the UV–Vis spectra of HP–COOH and HP–NH_2_ in the DMSO and DMF solutions, respectively. In Figure 3a, these two polymers display similar absorption behavior in the DMSO and DMF solutions. In the DMSO solution, HP–COOH has a maximum absorption peak at about 285 nm and a shoulder peak at around 336 nm. HP–NH_2_ shows a maximum absorption peak at about 283 nm and a shoulder peak at around 336 nm. In the DMF solution (Figure 3b), the maximum absorption peaks of HP–COOH and HP–NH_2_ are both at 280 nm. The shoulder peaks of HP–COOH and HP–NH_2_ are at around 334 and 342 nm, respectively. The absorptions in the short wavelength region (280–285 nm) are ascribed to *π*–π^*^ (π^*^ means the antibonding orbital) transition and the absorptions in the long wavelength region (333–342 nm) might be due to the intramolecular charge transfer.

**Figure 4:**
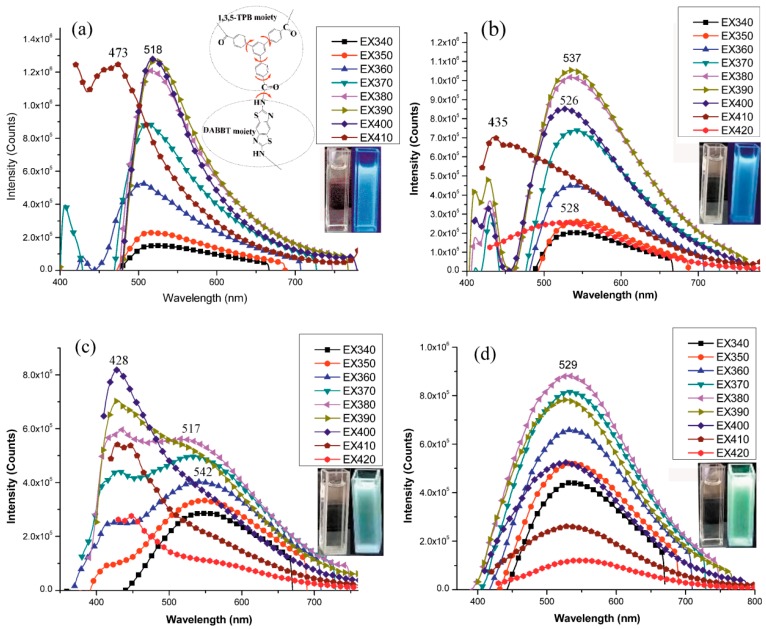
Fluorescence spectra of HP–COOH and HP–NH_2_ in DMSO and DMF solutions: (**a**) HP–COOH in DMF (c = 0.135 mg/mL); (**b**) HP–NH_2_ in DMF (c = 0.125 mg/mL); (**c**) HP–COOH in DMSO (c = 0.043 mg/mL); and (**d**) HP–NH_2_ in DMSO (c = 0.05 mg/mL).

The fluorescence spectra of HP–COOH and HP–NH_2_ in the DMSO and DMF solutions are shown in Figure 4. The insets in Figure 4 are the intramolecular rotation sketch maps of the two polymers and the photographs of the polymer solutions under both natural light and 365 nm. In the DMF solution, the maximum emission wavelengths (λ_em_) of HP–COOH and HP–NH_2_ are about 518 nm (green light, Figure 4a) and 537 nm (green light, Figure 4b), respectively. The Stokes shifts of HP–COOH and HP–NH_2_ in DMF solution are 128 nm (0.79 eV) and 147 nm (0.87 eV), respectively (see Table 2). The corresponding optimal excitation wavelengths (λ_ex_) of HP–COOH and HP–NH_2_ are about 400 and 390 nm, respectively. For HP–COOH in DMF solution, the maximum emission wavelength (λ_em_) changed from about 518 to 473 nm when the excitation wavelength was varied from 400 to 410 nm. This was the same for HP–NH_2_ in the DMF solution (Figure 4b). When the excitation wavelength was changed from 400 to 410 nm, the maximum emission wavelength changed from about 526 to 435 nm. When the excitation wavelength was moved from 410 to 420 nm, the emission intensity of HP–NH_2_ decreased rapidly, and the maximum emission wavelength changed from about 435 to 528 nm. Interestingly, there was a saltation in the fluorescence emissions of HP–COOH and HP–NH_2_ in the DMF solution when the excitation wavelength was changed from 400 to 410 nm. This may be ascribed to the macromolecular conformational changes [3,52,53] of the polymers that can be converted into one another merely by rotating the single bonds of the molecules when excited by a light with certain energy (as shown in Figure 4a). For the above two polymers, the critical excitation wavelength associated with the chain conformational changes might be 400 nm. In addition, there are moderate emissions in the wavelength range of 400 to 450 nm for both HP–COOH and HP–NH_2_ in DMF solutions, which may be ascribed to the intramolecular charge transfer between the donor and acceptor. It is the same for HP–COOH in DMSO solution (Figure 4c). For HP–NH_2_ in DMSO (Figure 4d), the emissions belonging to the intramolecular charge transfer are not obvious, which may be merged into the strong and broad emission of HP–NH_2_ in DMF.

In the DMSO solution (Figure 4c), HP–COOH had two emission peaks, one is in the relatively short wavelength region (about 428 nm, bluish violet light), and the other in the relatively long wavelength region (around 517 to 542 nm, green light). When the excitation wavelength increased from 340 to 380 nm, the fluorescence intensities at about 542 and 428 nm both increased. Emission in the relatively long wavelength region reached the highest value when excited by light of 380 nm. After that, the emission peak at about 517 nm decreased when the excitation wavelength was further increased to 390 and 400 nm. When excited at 400 nm, the long wavelength region emission disappeared, and the maximum emission changed to the relatively short wavelength region (about 428 nm). This also can be ascribed to the chain conformational changes of the polymers that can be converted into one another merely when excited by light with certain energy. In the DMSO solution, the critical excitation wavelength associated with the chain conformational changes might be 400 nm, which is the same as in the DMF solution. The Stokes shift of HP–COOH in DMSO is 137 nm (0.86 eV), as shown in Table 2.

For HP–NH_2_ in the DMSO solution (Figure 4d), when the excitation wavelength changed from 340 to 420 nm, HP–NH_2_ displayed a single and broad emission. The maximum emission wavelength (λ_em_) was at about 529 nm (green light) with an optimal excitation wavelength of 380 nm. The Stokes shift of HP–NH_2_ in DMSO (149 nm, 0.92 eV) and in DMF (147 nm, 0.87 eV) are larger than that of HP–COOH in DMSO (137 nm, 0.86 eV) and in DMF (128 nm, 0.79 eV), respectively, as shown in Table 2. For both HP–COOH and HP–NH_2_, the Stokes shift in DMSO is larger than that in DMF solutions, which can be ascribed to the polarity of DMSO is higher than that of DMF. 

**Figure 5:**
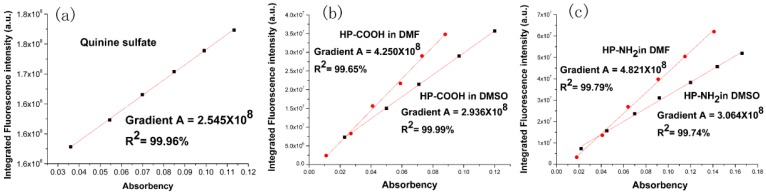
Linear plots for quinine sulfate standard samples and the two polymers: (**a**) quinine sulfate in 0.1 M H_2_SO_4_; (**b**) HP–COOH in DMF and DMSO; ns (**c**) HP–NH_2_ in DMF and DMSO.

For luminescent materials, QY represents one of the most fundamental and important properties that eventually determines the suitability of materials for applications in optical devices, analysis, bio-sensing, and fluorescence imaging [54,55]. In this work, the fluorescence QYs of the two polymers were obtained relative to quinine sulfate in 0.1 M H_2_SO_4_ (Ф_st_ = 0.55 [56]), which has a fixed and known fluorescence QY value, according to the following equation:

(2) $$\Phi_{x}\;={\;\Phi }_{\mathrm{st}}\left(\frac{Grad_{x}}{Grad_{\mathrm{st}}}\right)\left(\frac{\eta_{x}^{2}}{\eta_{\mathrm{st}}^{2}}\right)$$

where the subscripts ST and X denote the standard and test sample, respectively; Φ is the fluorescence quantum yield; *Grad* is the gradient from the plot of integrated fluorescence intensity vs. absorbance; and η is the refractive index of the solvent. The fluorescence spectra were measured on a Horiba scientific Fluoromax-4 spectrofluorometer using HPLC grade DMSO and DMF as the solvents at room temperature; excitation and emission slit widths of 5 nm were used. The UV–Vis absorption spectrum and fluorescent spectrum of quinine sulfate in 0.1M H_2_SO_4_ are shown in the Supplementary Materials (Figures S6 and S7). The fluorescence spectra of both the unknown and the standard samples were obtained under identical conditions. The absorbance and the integrated intensity of the emission spectra were used to calculate the QY. The linear plots for the quinine sulfate standard samples and the two polymers in the DMF or DMSO solutions are shown in Figure 5. The gradient for each sample is proportional to the fluorescence QY of that sample. Conversion to an absolute QY is achieved according to Equation (1). The results are listed in Table 2. The fluorescence QYs of HP–COOH in the DMSO and DMF solutions are 77.75% and 104.65%, respectively, and the fluorescence QYs of HP–NH_2_ in the DMSO and DMF solutions are 81.14% and 118.72%, respectively. The amino-terminated polymer (HP–NH_2_) has a relatively higher QY than the carboxyl-terminated polymer (HP–COOH) in the same solvent. This can be attributed to the end group effect of hyperbranched polymers as described in our previous study [39]. The amino group (–NH_2_) is an electron donating group and reduces the energy gap between the HOMO and the LUMO, which therefore promotes the corresponding electron transition. There are large numbers of carboxyl groups (–COOH) on the periphery of HP–COOH; –COOH is an electron-withdrawing group and thus weakens fluorescence emission [39]. The two polymers all have relative high QYs in the DMF and DMSO solutions, and in particular, the QYs exceed 100% in the DMF solution. This might be ascribed to the quantum cutting effect where the energy of one absorbed photon is transformed into two or more emitted photons that are usually doped with lanthanide ions [57,58,59].

**Figure 6:**
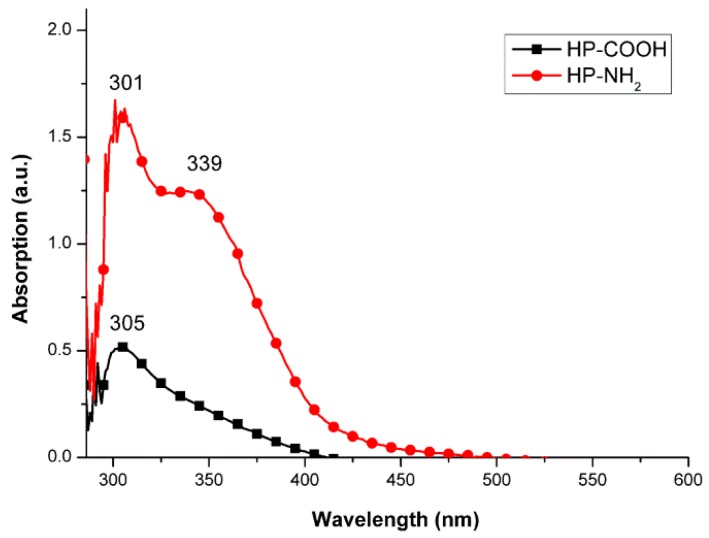
UV–Vis spectra of the HP–COOH and HP–NH_2_ films.

According to the above analysis, both HP–COOH and HP–NH_2_ have very high fluorescent QY in the DMSO or DMF solutions. Accordingly, the solid films of the two polymers should each have good fluorescent performance. Thus, the HP–COOH and HP–NH_2_ films were prepared using a solution evaporation method, and the optical properties of the prepared polymer films were studied. The SEM images of the HP–COOH and HP–NH_2_ films are shown in the Supplementary Materials (Figure S8). The UV–Vis spectra of the HP–COOH and HP–NH_2_ films are shown in Figure 6. HP–COOH has two absorption peaks at 302 and 308 nm. The maximum absorption is 308 nm. HP–NH_2_ shows a maximum absorption at 300 nm and a broad shoulder peak at 341 nm. The maximum absorption wavelength of the HP–COOH film is red-shifted 23 and 28 nm compared to the DMSO and DMF solutions, respectively. The maximum absorption wavelength of the HP–NH_2_ film is red-shifted 20 nm compared to the DMSO and DMF solutions. This might be attributed to the increased co-planarity of the hyperbranched polymer molecules in the solid films compared to the solutions. In solution, the two polymers exhibit a more twisted molecular conformation than in the solid film because of rotation about the single bonds of the macromolecules. Thus, the conjugation degree increases, and the maximum UV–Vis absorption is obviously red-shifted in the solid films compared to that in the DMF and DMSO solutions.

**Figure 7:**
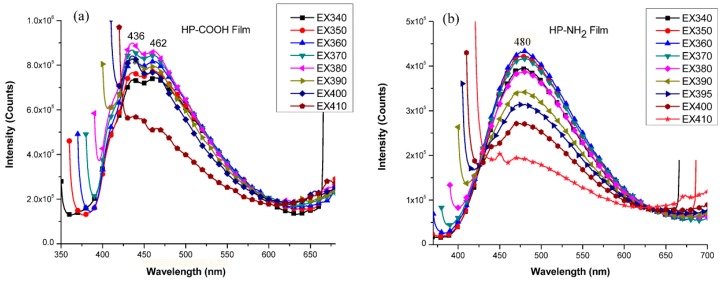
Fluorescence curves of: (**a**) HP–COOH; and (**b**) HP–NH_2_ in the solid films.

Figure 7 shows the fluorescence of the solid films of HP–COOH and HP–NH_2_. HP–COOH (Figure 7a) displays two emission peaks at about 436 and 462 nm (blue light) under the excitation wavelength of 340 to 410 nm. The maximum emission wavelength is 436 nm. The optimal excitation wavelength for HP–COOH is 380 nm. The maximum absorption of HP–COOH film is 305 nm, and so the Stokes shift of HP–COOH film is about 131 nm (0.42 eV), as shown in Table 2. When the excitation wavelength was increased from 340 to 380 nm, the emission intensities gradually increased. When the excitation wavelength was increased from 380 to 410 nm, the emission intensities decreased. In particular, when the excitation wavelength was increased from 400 to 410 nm, the fluorescence intensity reduced significantly. HP–NH_2_ exhibits a single fluorescence emission centered at about 480 nm (blue light), as shown in Figure 7b. The optimal excitation wavelength for HP–NH_2_ is 460 nm. The maximum absorption of HP–NH_2_ film is 300 nm, and so the Stokes shift of HP–NH_2_ film is about 179 nm (0.86 eV), as shown in Table 2, which is larger than that of HP–COOH film. The fluorescence quantum yields of the two hyperbranched polymers films were measured by Horiba scientific Fluoromax-4 spectrofluorometer with an integral sphere. The excitation wavelengths were 380 and 360 nm for HP–COOH and HP–NH_2_, respectively. The fluorescent QYs of HP–COOH and HP–NH_2_ films are 2.01% and 4.89%, respectively, which is much lower than that in DMSO or DMF solutions. This might be due to the aggregation-induced quenching effect (ACQ) [60]. The large effective Stokes shifts (0.42 to 0.92 eV) of the two polymers may be useful to minimize the self-absorption and light scattering in optical materials [61,62]. These two polymers are promising candidates for luminescent solar concentrators and blue light emitting materials.

**Figure 8:**
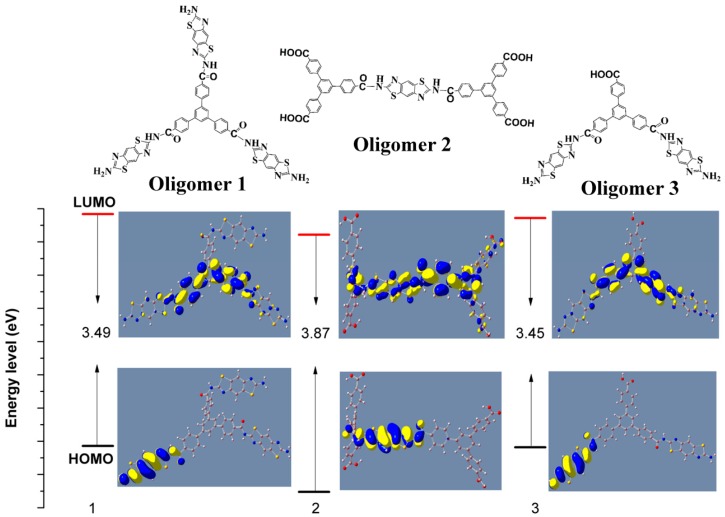
Visualization of HOMO, LUMO and energy gaps of three oligomers calculated by Gaussian 09 software package.

To understand the electronic structures of the two synthesized polymers, DFT calculations of three selected oligomers were performed by standard computational methods and basis sets using Gaussian 09 software (Gaussian Inc., Wallingford, the United States) package. The HOMO and LUMO wave functions and energy gaps of three selected oligomers are illustrated in Figure 8. Oligomer 1 represents the dendrimer unit (D, Scheme 1) or the terminal unit (T_2_, Scheme 1) of the hyperbranched polymers, in which the carboxyl groups of H_3_BTB are all reacted with amino groups of DABBT to form amide bond. The molecular weight of Oligomer 1 is 1051 g/mol. Oligomer 2 represents the terminal unit (T_1_, Scheme 1) of the hyperbranched polymers, in which the carboxyl groups is excessive and the amino groups are consumed to generate amide. The molecular weight of Oligomer 2 is 1063 g/mol. Oligomer 3 represents the linear unit (L, Scheme 1) of the hyperbranched polymers, in which two carboxyl groups of H_3_BTB are consumed and one carboxyl group remain. The molecular weight of Oligomer 3 is 847 g/mol. The HOMO is localized on the Pi orbital (*π*–orbital) of the DABBT unit for the three oligomers, while the LUMO is strongly localized on the *π*–orbital of the H_3_BTB unit for the three oligomers. The two hyperbranched polymers have twisted structure with DABBT as a donor unit and H_3_BTB as an acceptor unit. The HOMO and LUMO were effectively separated. The energy gaps were 3.49 eV (Oligomer 1), 3.87 eV (Oligomer 2), and 3.45 eV (Oligomer 3), respectively. The effective HOMO–LUMO separation helps to induce the intramolecular charge transfer transition from HOMO to LUMO [63,64]. There observations corroborate well with the UV–Vis absorption and fluorescence properties of HP–COOH and HP–NH_2_.

### 3.5. XRD Analysis

**Figure 9:**
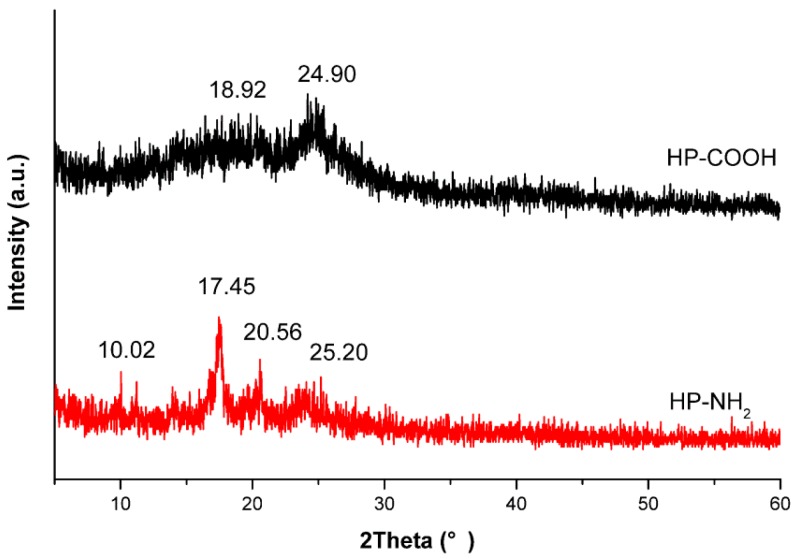
Wide angle X-ray diffraction (WAXD) spectra of HP–COOH and HP–NH_2_.

The wide angle X-ray diffraction (WAXD) spectra of HP–COOH and HP–NH_2_ are shown in Figure 9. HP–COOH has two weak and broad diffraction peaks at about 2θ = 18.92° and 24.90°, indicating that this polymer is almost amorphous with certain crystallinity. For HP–NH_2_, there are an intense and sharp peak at 2θ = 17.45° and three weak peaks at about 2θ = 10.02°, 20.56°, and 25.20°, illustrating that HP–NH_2_ is a semi-crystalline polymer.

### 3.6. Thermal Analysis

**Figure 10:**
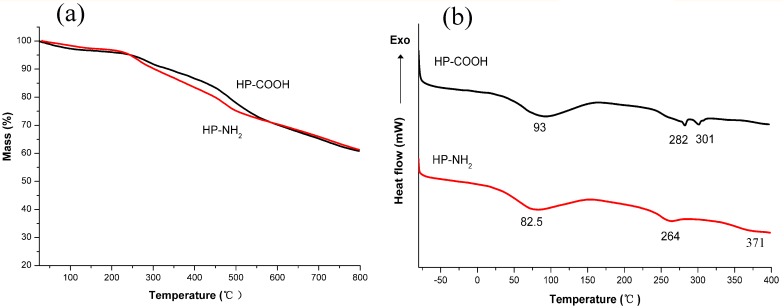
(**a**) TGA; and (**b**) DSC curves of HP–COOH and HP–NH_2_.

The TGA and DSC thermograms of HP–COOH and HP–NH_2_ are shown in Figure 10. The two polymers display similar thermal degradation behavior in the range of 30 to 800 °C. There are two stages in the TGA curves of HP–COOH and HP–NH_2_. The first stage is from 30 °C to about 282 °C, with weight losses of 6.95% and 8.57% for HB–COOH and HB–NH_2_, respectively. The weight losses in this stage were ascribed to the slow removal of the absorbed water from the hyperbranched polymers. The second stage is from about 282 °C to about 800 °C. The weight loss in this second stage is much higher than that in the first stage, and this is because of the decomposition of the synthesized polymers. The residual masses at 800 °C are about 60.8% and 61.3% for HB–COOH and HB–NH_2_, respectively. The thermal stability of HB–COOH is higher than that of HB–NH_2_ in the temperature range of 282 to about 583 °C. From 583 to about 800 °C, the thermal stability of HB–NH_2_ is slightly higher than that of HB–COOH. 

The DSC curves of HB–COOH and HB–NH_2_ display wide endothermic peaks at about 93 and 82.5 °C, respectively, and this is because of the glass transition of the synthesized polymers. The endothermic peaks centered at about 282 and 264 °C might be ascribed to the melting point (T_m_) of the crystallization regions of HP–COOH and HP–NH_2_, respectively, because both HP–NH_2_ and HP–COOH have certain crystallinity as displayed in their XRD curves (Figure 9). The endothermic peaks centered at about 301 and 371 °C might be ascribed to the thermal decomposition temperature (T_d_) of HP–COOH and HP–NH_2_, respectively. These two polymers have relatively high thermal stability under the temperature of 260 °C.

## 4. Conclusions

Two novel highly fluorescent hyperbranched polybenzobisthiazole amides with different terminal groups were synthesized via the A_2_ + B_3_ approach using H_3_BTB and DABBT as reaction materials, by regulating the mole ratio of the monomers. The prepared polymers were characterized using FTIR and ^1^H-NMR analysis. GPC analysis showed that the M_n_, M_w_, and PDI of HP–COOH and HP–NH_2_ were 7426 g/mol, 10,195 g/mol, and 1.37, respectively. The M_n_, M_w_, and PDI of HP–NH_2_ were 10,280 g/mol, 19,291 g/mol and 1.88, respectively. The two polymers are soluble in DMSO and DMF. Their solutions mainly emitted strong green light (517–537 nm) when the excitation wavelength varied from 340 to 410 nm. The relative fluorescence QYs in the DMSO solution were calculated as 77.75% and 81.14% with the Stokes shifts of 137 nm (0.86 eV) and 149 nm (0.92 eV) for HP–COOH and HP–NH_2_, respectively, using quinine sulfate as the standard. In the DMF solution, the relative fluorescence QYs of HP–COOH and HP–NH_2_ were calculated as 104.65% and 118.72%, with the Stokes shifts of 128 nm (0.79 eV) and 147 nm (0.87 eV), respectively. Their films mainly emitted strong blue light with the maximum emission wavelength of 436 nm and 480 nm for HP–COOH and HP–NH_2_, respectively. The Stokes shifts for HP–COOH and HP–NH_2_ films were 131 nm (0.42 eV) and 179 nm (0.86 eV), respectively. DFT calculations demonstrated that the HOMO and LUMO were effectively separated with DABBT as a donor unit and H_3_BTB as an acceptor unit. The effective HOMO–LUMO separation helps to induce the intramolecular charge transfer transition from HOMO to LUMO. The two hyperbranched polymers possess the merits of large Stokes shift and high fluorescence quantum yield, which may simultaneously solve the problems of nonradioactive re-absorption of many fluorescent materials, and may become promising candidates as luminescent solar concentrators for photovoltaic devices and blue light emitting materials.

## Figures and Tables

**Table 1:** GPC data of HP–COOH and HP–NH_2_.

Samples	Monomer Feed Ratio of A_2_ to B_3_	M_n_	M_w_	M_p_	M_z_	M_z+1_	PDI ^a^
HP–COOH	1:1	7426	10,195	12,314	13,387	16,547	1.37
HP–NH_2_	9:4	10,280	19,291	11,035	33,938	49,610	1.88

^a^ PDI represents polydispersity.

**Table 2:** Photophysical properties of HP–COOH and HP–NH_2_.

Samples	λ_abs_ (nm)	λ_em_ (nm)	Stokes Shift (nm/eV) ^a^	Eg^opt^ (eV) ^b^	Ф_FL_^c^
HP–COOH in DMSO	285, 336	427, 517	137/0.86	3.13	77.75%
HP–COOH in DMF	280, 333	518	128/0.79	2.55	104.65%
HP–COOH Film	305	436, 462	131/0.42	3.02	2.01%
HP–NH_2_ in DMSO	283, 336	529	149/0.92	2.97	81.14%
HP–NH_2_ in DMF	280, 342	537	147/0.87	2.76	118.72%
HP–NH_2_ Film	301, 339	480	179/0.86	2.55	4.89%

^a^ △E_Stokes_ is calculated by the following equation: ${\Delta E}_{\mathrm{Stokes}}\;=\;\frac{1240}{\lambda_{\mathrm{ex}}}\;-\;\frac{1240}{\lambda_{\mathrm{em}}}$; ^b^ optical energy gap (Eg^opt^) was calculated from the onset of UV–Vis absorption spectra, Eg^opt^ = 1240/λ; ^c^ Ф_FL_ represents the fluorescence quantum yields of hyperbranched polymer solutions calculated using quinine sulfate as the standard. The gradient for each sample is proportional to the fluorescence quantum yield of that sample. Conversion into an absolute quantum yield is achieved through the equation given in the text (Equation (1)). Ф_FL_ of polymer films were measured by Horiba scientific Fluoromax-4 spectrofluorometer with an integral sphere.

## Supplementary Materials

The following are available online at www.mdpi.com/2073-4360/9/8/304/s1, Figure S1: ^1^H–NMR spectrum of DABBT, Figure S2: ^13^C–NMR spectrum of DABBT, Figure S3: ^1^H–NMR spectrum of H_3_BTB, Figure S4: ^13^C–NMR spectrum of H_3_BTB, Figure S5: FTIR spectra of HP–COOH and HP–NH_2_, Figure S6: the UV–Vis absorption spectrum of quinine sulfate in 0.1 M H_2_SO_4_, Figure S7: the fluorescent spectrum of quinine sulfate in 0.1 M H_2_SO_4_, Figure S8: SEM images of: HP–COOH film (A,B); and HP–NH_2_ film (C,D).
