# Supplementary material for: Synthesis of Novel Hyperbranched Polybenzo-Bisthiazole Amide with Donor–Acceptor (D-A) Architecture, High Fluorescent Quantum Yield and Large Stokes Shift
Source: Polymers (Basel). 2017 Jul 25;9(8):304. doi: 10.3390/polym9080304 (PMC6418910; doi:10.3390/polym9080304)
Supplement: Supplementary file 1 [file polymers-09-00304-s001.pdf]

Supplementary Materials

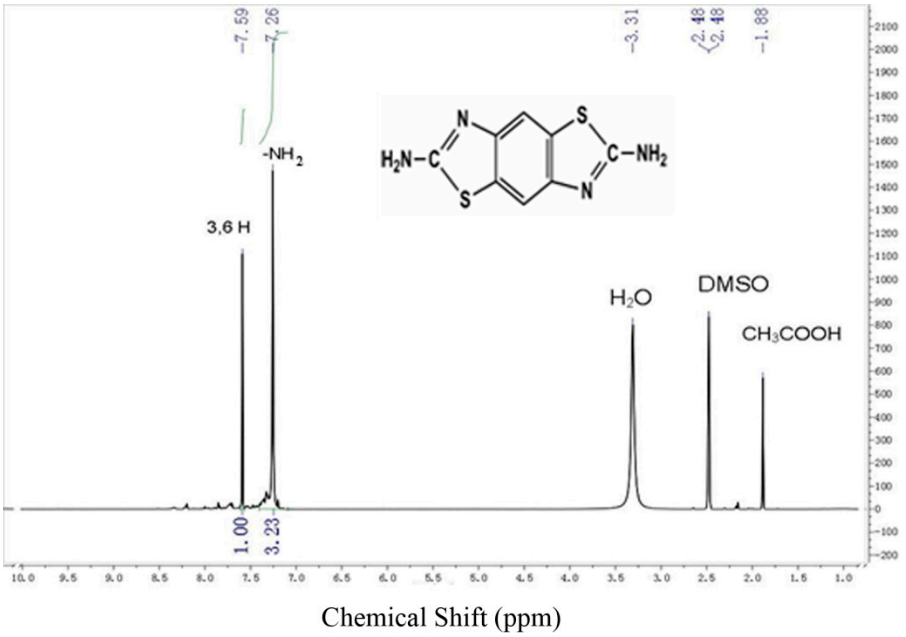

Figure S1. ^{1}H NMR spectrum of DABBT.

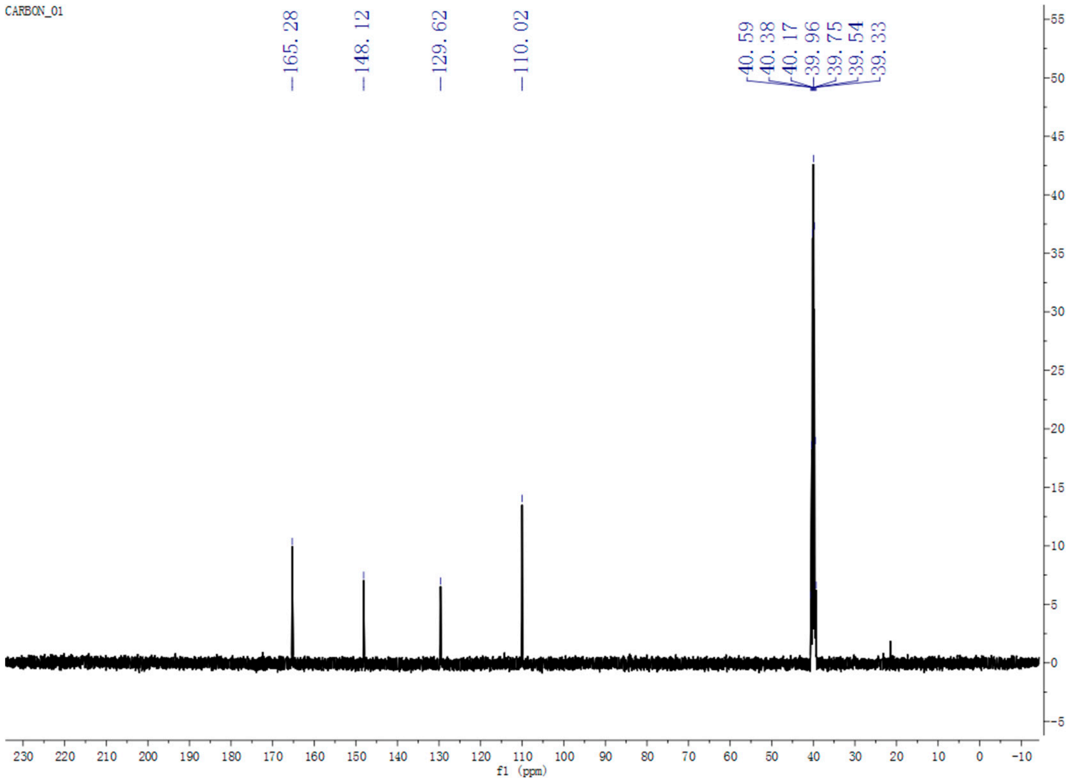

Figure S2. ^{13}C NMR spectrum of DABBT.

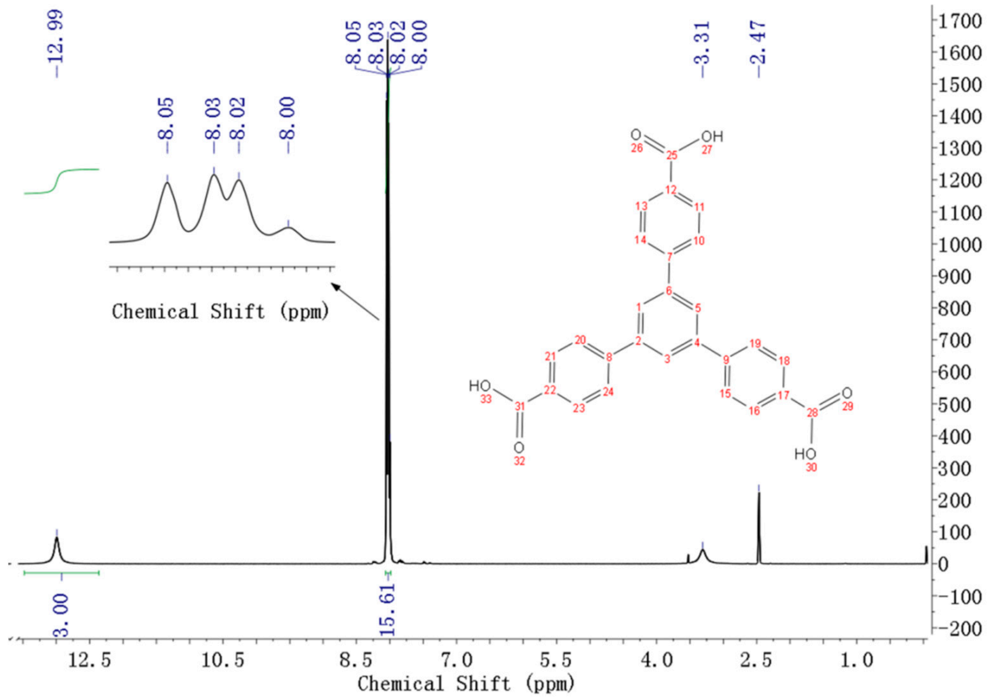

Figure S3. ^{1}H NMR spectrum of H_3BTB.

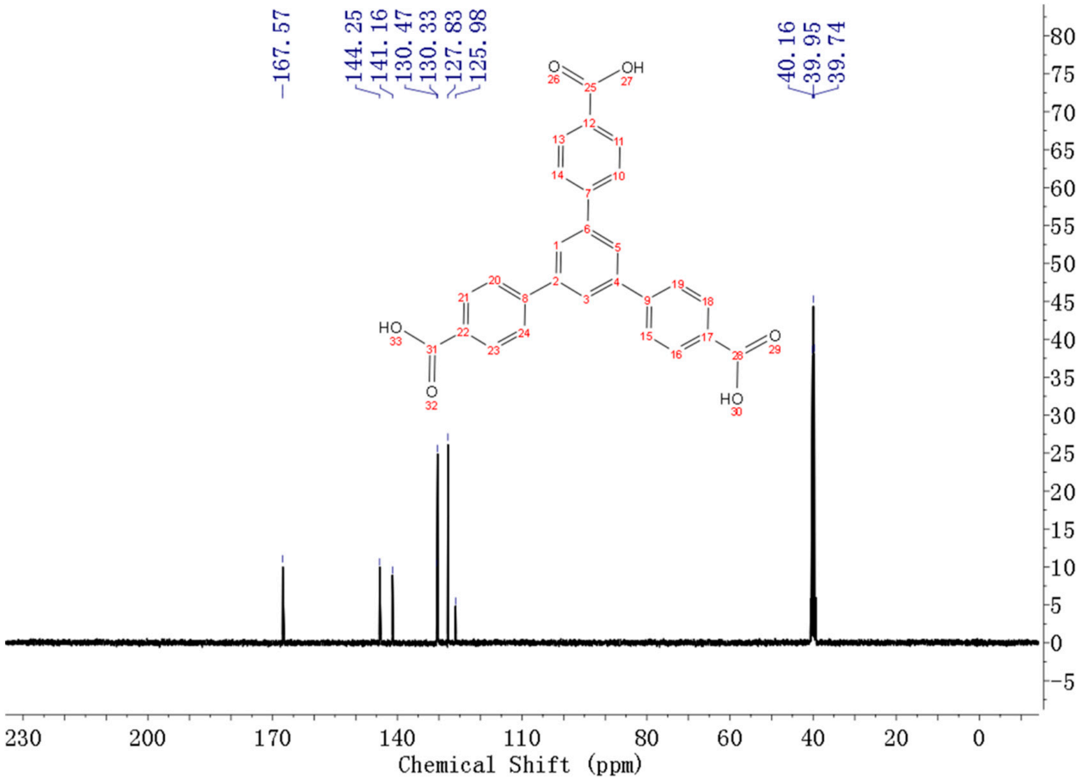

Figure S4. ^{13}C NMR spectrum of H_3BTB.

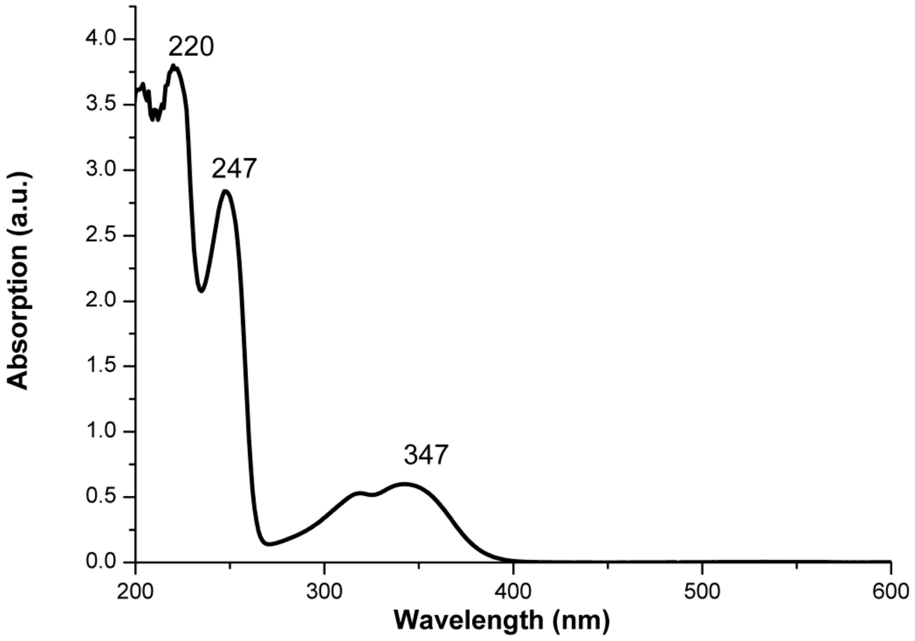

Figure S5. UV-vis absorption spectrum of quinine sulfate in 0.1M H_2SO_4.

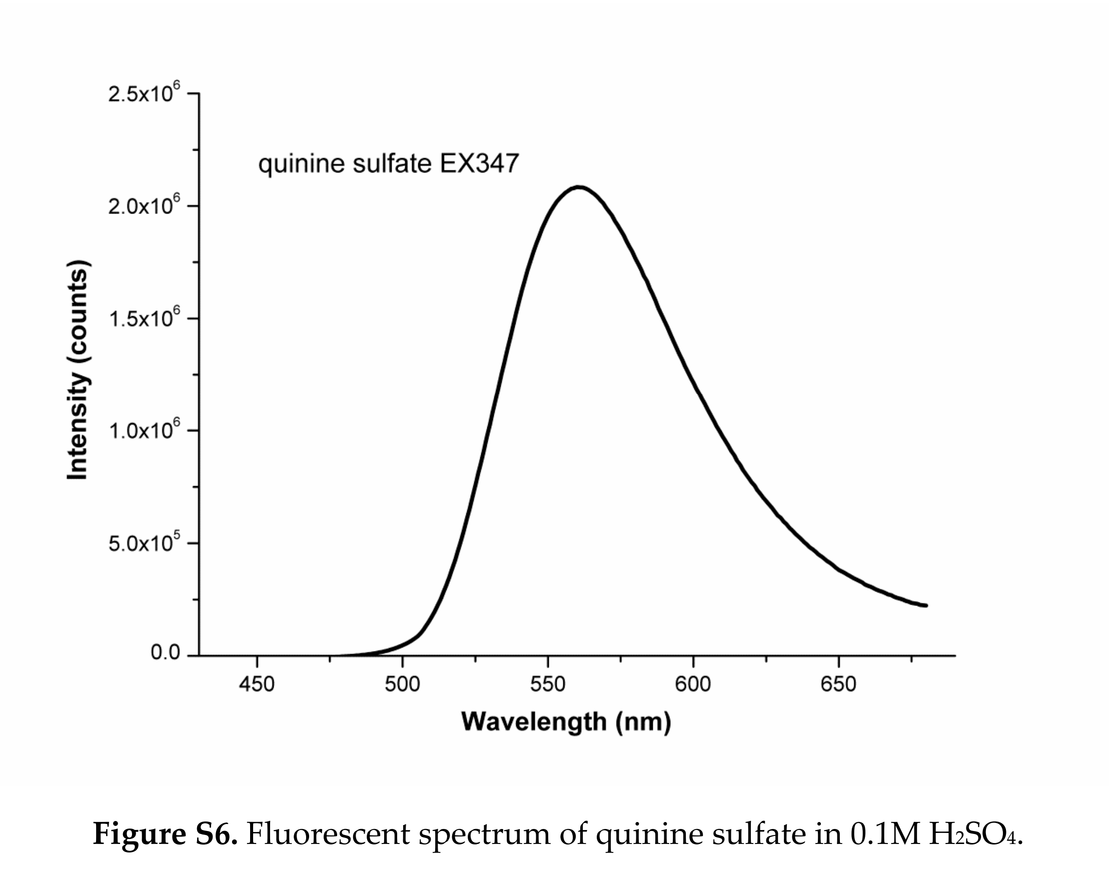

Figure S6. Fluorescent spectrum of quinine sulfate in 0.1M H_2SO_4.

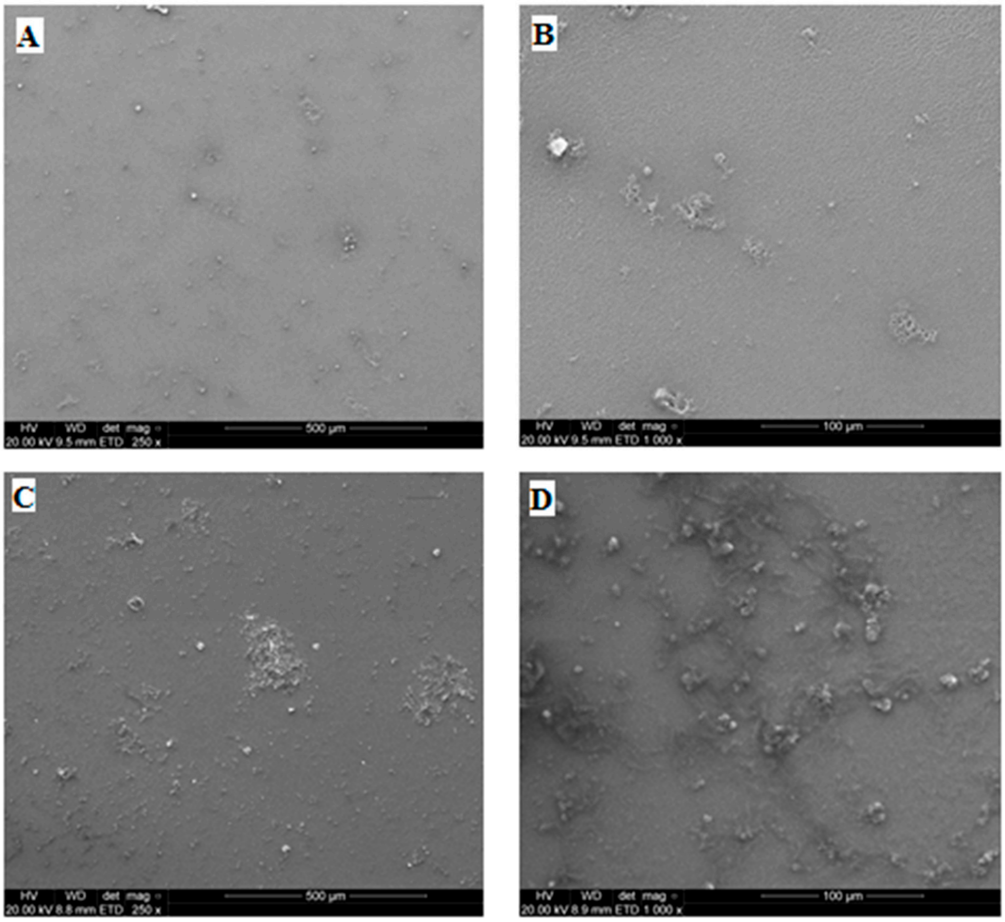

Figure S7. SEM images of HP-COOH (A, B) and HP-NH_2 (C, D) films.
